# Disinfection by hydrogen peroxide nebulization increases susceptibility to avian pathogenic *Escherichia coli*

**DOI:** 10.1186/s13104-015-1329-z

**Published:** 2015-08-27

**Authors:** Leon H. Oosterik, Huruma N. Tuntufye, Steven Janssens, Patrick Butaye, Bruno M. Goddeeris

**Affiliations:** Department of Biosystems, KU Leuven, Kasteelpark Arenberg 30, 3001 Louvain, Belgium; Department of General Bacteriology, CODA-CERVA, Groeselenberg 99, 1180 Brussels, Belgium; Department of Veterinary Microbiology and Parasitology, Sokoine University of Agriculture, P.O. 16 Box 3019, Chuo Kikuu, 67125 Morogoro, Tanzania; Department of Pathology, Bacteriology and Poultry Diseases, Ghent University, Salisburylaan 133, 9820 Merelbeke, Belgium; Department of Virology, Parasitology and Immunology, Faculty Veterinary Medicine, Ghent University, Salisburylaan 133, 9820 Merelbeke, Belgium; Department of Biosciences, Ross University, P.O. Box 335, Basseterre, St Kitts West Indies

**Keywords:** Hydrogen peroxide, Avian pathogenic *Escherichia coli*, Disinfection

## Abstract

**Background:**

Avian pathogenic *Escherichia coli* (APEC) are the major cause of economic losses in the poultry industry worldwide. Traditionally, antibiotics are used to treat and prevent colibacillosis in broilers. Due to resistance development other ways of preventing/treating the disease have to be found. Therefore during this study the nebulization of low concentrations of hydrogen peroxide (H_2_O_2_) was tested in the presence of chickens to lower pathogenicity of APEC.

**Results:**

Significantly higher total lesion scores and higher *E. coli* concentrations were found in the spleen of chickens exposed to 2 % H_2_O_2_ compared to those exposed to 1 % H_2_O_2_ and control chickens which had been exposed to nebulization with distilled water. Higher total lesions scores and *E. coli* concentrations in the spleen were found in chickens exposed to 1 % H_2_O_2_ in comparison to control chickens (not significant).

**Conclusion:**

H_2_O_2_ is rendering animals more prone to APEC infection contraindicating H_2_O_2_ nebulization in the presence of chickens.

## Background

Avian pathogenic *Escherichia coli* (APEC) causes colibacillosis in chickens of all ages, leading yearly to major losses to the poultry industry [1, 2]. Antibiotics have been used for a long time to treat and prevent diseases in chickens; with the consequence of antibiotic resistance development posing a huge threat to the public health worldwide [3] and transfer of virulence and resistance genes to other bacteria [4–6]. Sanitation and cleaning is a first way to reduce infection pressure [7], but is normally performed between different flocks in the barn to prevent flock to flock transmission. The information about the use of disinfectants in the presence of animals to lower pathogenicity of APEC is limited. On conditions safe to the animals, nebulization of hydrogen peroxide (H_2_O_2_) in the presence of chickens may possibly lower pathogenicity of APEC, since (pathogenic) *E. coli* is normally present in high concentrations in the barn in the form of faeces-contaminated dust [1, 2, 8]. Predisposing factors, such as viral live vaccines, render chickens more prone to APEC infection [9, 10]. Several studies on H_2_O_2_ toxicity have been performed on rats, mice, rabbits and dogs but there is no documentation on such studies in chickens. As toxicity is depending on many factors like exposure time, number of exposures and concentration [11] the goal of this study was to test if H_2_O_2_ nebulization in the presence of chickens can lower pathogenicity of APEC.

## Methods

The animal experiment was approved by the ethical committee of the KU Leuven (Permit Number: P176/2011) and conducted in strict accordance with the Federation of European Laboratory Animal Science Associations guidelines.

Fifteen one-day-old chickens (Ross; Belgabroed NV, Merksplas, Belgium) were housed in a disinfected barn and provided with water and feed ad libitum. At 4 weeks of age, the chickens were orally vaccinated against Newcastle disease (10^6^ mean egg infective dose; MSD Animal Health, Boxmeer, the Netherlands) to render them subsequently more susceptible to APEC challenge. Three days post vaccination (dpv), the chickens were transferred and equally divided over three disinfected isolators (dimensions: 1.5 × 1.0 × 1.0 m, volume ≈ 1.6 m^3^). Three and 4 dpv they were aerogenically infected with a virulent APEC strain CH2 [12] (10 ml of 10^10^ colony forming units/ml (cfu/ml)) with the help of a compressor (N 811 KN.18; KNF Neuberger, Aarselaar, Belgium) and nebulizer (Cirrus 2; Intersurgical, Uden, the Netherlands). The chickens were exposed to the aerosols for 60 min. One day thereafter or 5 dpv they were exposed to 10 ml of different H_2_O_2_ concentrations (EcoClearProx^®^, ABT Belgium BVBA, Affligem, Belgium) by nebulization for 60 min [Group 1—2 % H_2_O_2_; Group 2—1 % H_2_O_2_; Group 3—distilled water (DW)].

Eleven dpv all surviving chickens were euthanized by cervical dislocation for clinical necropsy. Macroscopic lesions were scored according to Vandemaele et al. [12].

From two or three chickens per group (chickens that had macroscopic lesions) the spleen was aseptically removed and homogenized in sterile phosphate buffered saline (PBS) (1 g of tissue in 1 ml PBS) as described before [13, 14]. Ten-fold dilutions were plated on MacConkey agar (Oxoid CM0115; Erembodegem, Belgium) and incubated at 37 °C for 24 h, after which bacterial numbers (cfu/g) were determined.

Results were analyzed with SAS software version 9.4 (SAS Institute Cary, North Carolina, USA) and R [15]. The sum of the total lesion score and the lesion scores per organ were calculated for the three groups. To evaluate the hypothesis of a higher total lesion score in infected chickens treated with DW than H_2_O_2_, the total lesion scores were analyzed by means of an ordinal logistic regression model. Results of ordinal logistic regression are presented as the odds ratio of having a high lesion score. Average bacterial concentrations were compared using the Kruskal–Wallis Test for significant differences between multiple groups and the Wilcoxon Rank Sum Test for differences between each pair of groups. *P* ≤ 0.05 was considered significant.

## Results and discussion

Mortality and lesions scores of infected/treated chickens are given in Table 1. None of the chickens died after infection. Chickens that were exposed to H_2_O_2_ had a higher sum of total lesion scores (Group 1: 42 and Group 2: 20) than chickens exposed to DW (Group 3: 14). Infected chickens treated with 1 % H_2_O_2_ had a 3.53 (95 % CI, 0.30–88.84) higher chance of developing a high lesion score (score: 14) than infected chickens treated with DW, while this chance was 12.28 (95 % CI, 1.01–357.07) higher in infected chickens treated with 2 % H_2_O_2_ than with 1 % H_2_O_2_. Thus nebulization of 2 % H_2_O_2_ does increase the lesion scores.

**Table 1:** Mortality and lesion scores of infected/treated chickens

Group	Infection^a^	Treatment^b^	Total^c^	Deaths^d^	Sum of lesion scores							
					Liver	PRD	TAS-L	TAS-R	LU-L	LU-R	AAS	TOT
1	CH2	2 % H_2_O_2_	5	0	6	6	6	6	6	6	6	42^A^
2	CH2	1 % H_2_O_2_	5	0	2	2	4	4	2	2	4	20^B^
3	CH2	DW	5	0	2	2	2	2	2	2	2	14^B^

*PRD* pericardium, *TAS-L* thoracic air sac-left, *TAS-R* thoracic air sac-right, *LU-L* lung-left, *LU-R* lung-right, *AAS* abdominal air sacs, *TOT* total lesion score and *DW* = distilled water

^a^Aerogenic infection with 10 mL 10^10^ cfu/ml APEC CH2 at 31 and 32 days of age

^b^Chickens aerogenically treated with 2 % H_2_O_2_, 1 % H_2_O_2_ or distilled water, at 33 days of age i.e. 24 h after the 2nd infection

^c^Total number of chickens per group

^d^Total number of chickens that died or were euthanized during the experiment

Numbers within the same column with different capital letters show significant differences (*P* ≤ 0.05)

**Fig. 1:**
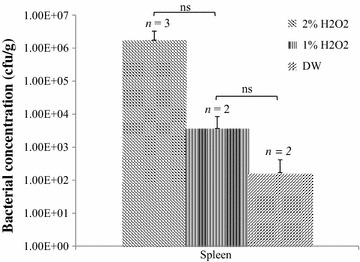
Isolated bacterial concentrations from the spleen. *cfu* colony forming units, *DW* distilled water, *ns* not significant (*P* ≥ 0.05)

Bacterial numbers (cfu/g) isolated from the spleen in chickens of Group 1, Group 2 and Group 3 are shown in Fig. 1. The highest bacterial load from the spleen was obtained in the group of chickens exposed to aerosols of 2 % H_2_O_2_, followed by 1 % H_2_O_2_ and DW (*P* = 0.11). However, the results are not significant, most likely due to the low number of tested chickens during this study.

This worsening effect after H_2_O_2_ nebulization is probably due to the caustic effect of H_2_O_2_ radicals on (ciliated) epithelial cells, such as the case for Newcastle disease virus infection (or vaccination with virulent vaccines) in one-day-old chickens [16], making it easier for APEC to cross the upper respiratory tract causing colibacillosis. The presented results give a good indication that nebulization of H_2_O_2_ (higher than 1 %) in the presence of chickens is not advisable. As toxicity was also shown to be dependent on exposure time and number of exposures (Anonymous 2003), these parameters should also be taken into consideration, during future studies. H_2_O_2_ concentrations lower than 1 % can, however, be used in an experimental respiratory infection model to predispose the chickens to APEC infection.

## Conclusion

H_2_O_2_ nebulization in the presence of chickens in order to lower pathogenicity of APEC is not advisable, since it makes chickens more susceptible to APEC infection.

## References

[1] Barnes HJ, Nolan LK, Vaillantcourt JP, Saif YM (2008). Colibacillosis. Diseases of poultry.

[2] Dziva F, Stevens MP (2008). Colibacillosis in poultry: unravelling the molecular basis of virulence of avian pathogenic *Escherichia coli* in their natural hosts. Avian Pathol.

[3] Singer RS, Hofacre CL (2006). Potential impacts of antibiotic use in poultry production. Avian Dis.

[4] Ewers C, Li G, Wilking H, Kiessling S, Alt K, Antáo EM, Laturnus C, Diehl I, Glodde S, Homeier T (2007). Avian pathogenic, uropathogenic, and newborn meningitis-causing *Escherichia coli*: how closely related are they?. Int J Med Microbiol.

[5] Johnson JR, Murray AC, Gajewski A, Sullivan M, Snippes P, Kuskowski MA, Smith KE (2003). Isolation and molecular characterization of nalidixic acid-resistant extraintestinal pathogenic *Escherichia coli* from retail chicken products. Antimicrob Agents Chemother.

[6] Johnson JR, Delavari P, O’Bryan TT, Smith KE, Tatini S (2005). Contamination of retail foods, particularly turkey, from community markets (Minnesota, 1999-2000) with antimicrobial-resistant and extraintestinal pathogenic *Escherichia coli*. Foodborne Pathog Dis.

[7] Gehan MG, Anwer W, Amer HM, IM W, Rezk A, Badawy EM (2009). In vitro efficacy comparisons of disinfectants used in the commercial poultry farms. Int J Poult Sci.

[8] Dho-Moulin M, Fairbrother JM (1999). Avian pathogenic *Escherichia coli* (APEC). Vet Res.

[9] Nakamura K, Ueda H, Tanimura T, Noguchi K (1994). Effect of mixed live vaccine (Newcastle disease and infectious bronchitis) and *Mycoplasma gallisepticum* on the chicken respiratory tract and on *Escherichia coli* infection. J Comp Pathol.

[10] La Ragione RM, Woodward MJ (2002). Virulence factors of *Escherichia coli* serotypes associated with avian colisepticaemia. Res Vet Sci.

[11] Anonymous: European Union risk assessment report hydrogen peroxide. In: Munn SJ, Allanou R, Aschberger K, Berthault F, De Bruijn J, Musset C, O’Connor S, Pakalin S, Paya-Perez A, Pellegrini G et al, vol 38. Luxembourg: Office for Official Publications of the European Communities; 2003. p 246.

[12] Vandemaele F, Ververken C, Bleyen N, Geys J, D’Hulst C, Addwebi T, van Empel P, Goddeeris BM (2005). Immunization with the binding domain of FimH, the adhesin of type 1 fimbriae, does not protect chickens against avian pathogenic *Escherichia coli*. Avian Pathol.

[13] Tuntufye HN, Lebeer S, Gwakisa PS, Goddeeris BM (2012). Identification of avian pathogenic *Escherichia coli* genes that are induced in vivo during infection in chickens. Appl Environ Microbiol.

[14] Tsonos J, Oosterik LH, Tuntufye HN, Klumpp J, Butaye P, De Greve H, Hernalsteens J-P, Lavigne R, Goddeeris BM (2014). A cocktail of in vitro efficient phages is not a guarantee for in vivo therapeutic results against avian colibacillosis. Vet Microbiol.

[15] R Development Core Team: R: A language and environment for statistical computing. R Foundation for Statistical Computing. http://www.R-project.org (2015). Last visited: January 2015.

[16] Mast J, Nanbru C, van den Berg T, Meulemans G (2005). Ultrastructural changes of the tracheal epithelium after vaccination of day-old chickens with the La sota strain of Newcastle disease virus. Vet Pathol Online.

